# Psychiatric co-morbidity among alcohol dependants

**DOI:** 10.4103/0019-5545.43058

**Published:** 2005

**Authors:** N. Heramani Singh, S.G. Sharma, Angelia M. Pasweth

**Affiliations:** * Associate Professor; ** Professor and Head; *** Postgraduate Student

**Keywords:** Alcohol dependence, psychiatric co-morbidity

## Abstract

**Background:**

Psychiatric disorders are common in alcoholics and such patients have a poorer prognosis.

**Aim:**

To determine the prevalence of psychiatric co-morbidity among alcohol-dependent subjects and to compare the prevalence of specific psychiatric disorders between them and a control group.

**Methods:**

The study assessed the prevalence of psychiatric co-morbidity in 100 alcohol-dependent subjects and 100 controls. A semi-structured proforma was used to record the sociodemographic variables and the history of alcohol abuse. Statistical analysis was done using the chi-square test.

**Results:**

The prevalence rate of psychiatric co-morbidity in alcohol-dependent subjects and controls was found to be 92% and 12%, respectively. The most common disorders were depression, antisocial personality disorder (ASPD) and phobia. There was a significant difference in the prevalence of psychiatric co-morbidity between alcohol-dependent subjects and controls.

**Conclusion:**

The findings indicate the need for an active consultation service for better insight into the prevention, treatment and outcome of alcohol dependence.

## INTRODUCTION

The prevalence of other psychiatric disorders in alcohol dependants is of concern to both clinicians and researchers. The issue of co-morbidity has now assumed centre-stage in psychiatric research, which has led investigators to comment that it may be one of the most important advancements in psychiatric nosology in the twentieth century.^1^ Alcohol dependence is often associated with other psychiatric disorders in clinical and community samples of alcoholics.^2^,^3^ Affective disorders (mainly depression), personality disorders (antisocial) and anxiety disorders (phobia) are widespread among persons who abuse alcohol. Recently gathered evidence suggests that having an additional psychiatric diagnosis has implications for the prognosis and treatment of alcohol dependence and the associated psychopathology.^4^,^5^

In a study by Tyndel,^6^ it was found that all the 100 patients who were hospitalized for alcoholism could be assigned another psychiatric diagnosis according to DSM-II. Most patients (58%) had a diagnosis of neurotic disorder. The other diagnoses were psychotic disorder (6%), personality disorder (36%), affective psychoses (4.2%), schizophrenia (1.3%) and paranoid disorder (0.5%).

Cadoret and Winokur^7^ examined 259 alcoholics and found that 101 (39%) suffered from depression. Lotufo-Neto and Gentil^8^ reported that although 26% of the clinic sample comprising alcoholics met the Clinician Rating Scale Criteria for agoraphobia and 20% for social phobia, most of these patients showed only mild symptoms and merely avoided a few phobic situations.

Morgenstern *et al.*^9^ studied the co-morbidity of alcoholism and personality disorder in a clinical population of 366 subjects. They found that 22.7% had an antisocial personality disorder (ASPD) and also reported a high prevalence rate for other types of personality disorders. Thus, it may be assumed that alcohol use and abuse has reached such an alarming degree that we should contemplate effective measures to discourage excessive drinking and identify the affected and vulnerable segments of our society.

The psychiatric co-morbidity was assessed in alcohol-dependent subjects and compared with a control group. The need for active intervention to prevent and treat alcohol dependence and improve the outcome of treatment was also assessed.

## METHODS

The study was conducted in a general hospital psychiatry unit (GHPU) setting. The index group included 100 subjects who fulfilled the DSM-IV criteria^10^ for alcohol dependence syndrome attending the Psychiatry OPD services at the Regional Institute of Medical Sciences (RIMS), Manipur. Hundred MBBS students were included as controls in the study after carefully excluding the presence of psychiatric/ organic diseases and substance use disorders.

A semi-structured proforma was used to record the sociodemographic variables and the history of alcohol abuse. After 2 weeks, all the patients were administered the Present State Examination (PSE) for a DSM-IV diagnosis. Finally, a thorough physical examination and investigations, if necessary, were done. Statistical analysis was done using the chi-square test.

## RESULTS

### Sociodemographic characteristics

Most of the subjects (43) belonged to the age group of 36–45 years. A large number were married (83%); 13% were unmarried and 2% each were divorced and widowers. Of the total, 29% were graduates. With respect to employment, 47% of the subjects were employed, 41% were businessmen and 12% were unemployed. Regarding income range per month, 57% earned from Rs 1000 to Rs 5000, 21% less than Rs 1000, 19% from Rs 5000 to Rs 10,000 and 3% earned more than Rs 10,000.

### Characteristics of alcohol-dependent subjects

The maximum number of subjects (35%) started using alcohol at the age of 16–20 years, 27% at the age of 21–25 years and 14% after the age of 26 years. Most subjects (79%) had developed dependence on alcohol before the age of 36 years, 44% between the ages of 15 and 25 years, 35% between the ages of 26 and 35 years and 25% after the age of 36 years. The majority of subjects (54%) were dependent on alcohol for 5 years or less (19% for >15 years, 16% for 6–10 years and 11% for 11–15 years).

The difference in the age of starting to take alcohol in the subjects was statistically significant (χ^2^=9.04, p<0.05); the difference in the age of development of the dependence syndrome was also significant (χ^2^=8.06, p<0.05). The number of years of dependence on alcohol was also tested. The χ^2^ value was found to be 46.16, thus the distribution is highly significant at the 1% probability level.

As many as 92% of alcohol-dependent subjects had co-morbid psychiatric disorders while only 12% in the control group had any psychiatric illness. This difference was found to be statistically highly significant (χ^2^=71.56, p<0.01).

Table 1 gives the distribution of psychiatric co-morbidity among alcohol-dependent subjects and those in the control group. The co-morbid disorders diagnosed were depression, panic disorder, generalized anxiety disorder (GAD), ASPD, other substance abuse, sexual dysfunction, obsessive–compulsive disorder (OCD) and schizophrenia.

**Table 1:** Comparison of psychiatric co-morbidity between alcohol-dependent subjects and the control group

Psychiatric co-morbidity	Alcohol-dependent subjects	Control group	χ^2^ value	p value	Remarks
Depression	26	4	16.13	p<0.01	Highly significant
Phobia	16	—	—	—	—
ASPD	21	—	—	—	—
Panic disorder	5	3	0.50	p<0.05	Insignificant
GAD	8	5	0.35	p<0.05	Insignificant
Other substance abuse	8	—	—	—	—
Sexual dysfunction	4	—	—	—	—
Schizophrenia	2	—	—	—	—
OCD	2	—	—	—	—

ASPD: Antisocial Personality Disorder; GAD: Generalized Anxiety Disorder; OCD: Obsessive-Compulsive Disorder

In the index group, depression was the most common diagnosis (26%), followed by ASPD (21%) and phobia (16%). The variation among the cases was highly significant (χ^2^=59.64, p<0.01). So the development of different psychiatric co-morbidity among alcohol-dependent subjects is evident. Only 12 in the control group had a psychiatric co-morbid condition; most of them had depression, panic disorder and GAD.

The chi-square test in patients with depression in both the groups was significant (χ^2^=16.13, p<0.01), whereas it was insignificant in cases with panic disorder and GAD. Thus, it may be concluded that none of the specific disorders are more prevalent than others in both the groups.

## DISCUSSION

This study focused on determining the prevalence of psychiatric co-morbidity among alcohol-dependent subjects. It also compared the prevalence of specific psychiatric disorders between the index and the control groups.

Unlike other studies conducted in a general hospital setting or other alcoholism treatment centres, all the subjects were males in this study. The reason was that alcohol consumption by women is socially unacceptable in this region and women may not avail of treatment openly in a general hospital setting. Moreover, more men than women use alcohol, and the ratio of men to women for an alcohol-related diagnosis is about 2:1 or 3:1.^11^

Graduate, married males in the age group of 36–45 years who are employed and earn less than Rs 5000/month comprise the most vulnerable group for alcohol dependence.

In this sudy, 62% of the subjects started consuming alcohol between the ages of 16 and 25 years, which is comparable to the study by Ross *et al.*^11^ in which most started alcohol consumption at around the age of 16±3.8 years. Forty-four per cent of the subjects developed dependence between the ages of 15 and 25 years, which shows that they had not only started early but also developed dependency while quite young. Fifty-four per cent of the subjects were dependent on alcohol for a period of 1–5 years and 19% for more than 15 years.

In the index group, psychiatric co-morbidity was found in 92% of the subjects which can be compared with the findings of other workers (range: 78%–100%).^6^,^11^ The most common psychiatric co-morbidity was depression (26%); this can be compared with the findings of Cadoret *et al.*^7^ (39%) and Alec *et al.*^12^ (33%). Phobia was diagnosed in 16% of the patients. Different types of phobias such as agoraphobia, social phobia, specific phobia or other phobic disorders were grouped together. The prevalence of phobia in most other studies^8^,^12^ was higher (range: 20%–29%); this might be because their study samples included female subjects and some of the samples were taken from anxiety clinics only. Among alcohol-dependent subjects, 21% had ASPD, which is comparable to the findings of Morgenstern *et al.*^9^(22.7%).

In the control group, only 12% had psychiatric co-morbidity; depression was diagnosed in 4%; GAD in 5% and panic disorders in 3%.

When the specific psychiatric disorders were compared among themselves, it was found that these disorders were more prevalent among alcohol-dependent subjects than in the control group. Therefore, the present study seems to have implications for better insight into the prevention, treatment and outcome of alcohol dependence.

## References

[1] Sabshin M (1991). Comorbidity. A central concern of psychiatry in the 1990s. Hosp Community Psychiatry.

[2] Weissman MM, Myers JK, Harding PS (1980). Prevalence and psychiatric heterogeneity of alcoholism in a United States urban community. J Stud Alcohol.

[3] Powell BJ, Penick EC, Othmer E (1982). Prevalence of additional psychiatric syndromes among male alcoholics. J Clin Psychiatry.

[4] Schuckit MA (1985). The clinical implications of primary diagnostic groups among alcoholics. Arch Gen Psychiatry.

[5] Mueller TI, Lavori PW, Keller MB (1994). Prognostic effect of the variable course of alcoholism on the 10-year course of depression. Am J Psychiatry.

[6] Tyndel M (1974). Psychiatric study of 100 alcoholic patients. Can J Psychiatry.

[7] Cadoret R, Winokur G (1974). Depression in alcoholism. Ann N Y Acad Sci.

[8] Lotufo-Neto F, Gentil V (1994). Alcoholism and phobic anxiety—a clinical—demographic comparison. Addiction.

[9] Morgenstern J, Langenbucher J, Labouvie E (1997). The comorbidity of alcoholism and personality disorders in a clinical population: Prevalence rates and relation to alcohol typology variables. J Abnorm Psychol.

[10] American Psychiatric Association (1994). Diagnostic and statistical manual of mental disorders.

[11] Ross HE, Glaser FB, Germanson T (1988). The prevalence of psychiatric disorders in patients with alcohol and other drug problems. Arch Gen Psychiatry.

[12] Alec R, Judith DJ, Danuta L (1991). Depression among alcoholics. Arch Gen Psychiatry.

